# Tigecycline pharmacokinetics in critically ill patients on renal replacement therapy: possible warnings and potential perspectives for the research agenda

**DOI:** 10.1186/s13613-020-00759-4

**Published:** 2020-10-14

**Authors:** Salvatore Lucio Cutuli, Gennaro De Pascale, Lucia Lisi, Simone Carelli, Pierluigi Navarra, Massimo Antonelli

**Affiliations:** 1 Dipartimento di Scienza dell’Emergenza, Anestesiologiche e della Rianimazione – UOC di Anestesia, Rianimazione, Terapia Intensiva e Tossicologia Clinica, Fondazione Policlinico Universitario A. Gemelli IRCCS, 00168 Rome, Italy; 2 Istituto di Anestesia e Rianimazione, Università Cattolica del Sacro Cuore, Rome, Italy; 3 Institute of Farmacologia, Università Cattolica del Sacro Cuore, L.go F. Vito 1, Rome, Italy

To the editor:

Honore et al. [1] suggested important insights into papers on tigecycline pharmacokinetics/pharmacodynamics in critically ill patients [2, 3], which warrant further considerations.

Eleven patients (34.4%) included in our trial [3] underwent continuous renal replacement therapy with low-adsorptive high-flux membranes (acrylonitrile, AN69), which was set in continuous standard volume veno-venous hemodiafiltration (CVVHDF, effluent dose prescribed 35 ml/kg/min) modality and used regional citrate anticoagulation. Although Broeker et al. [2] did not use regional citrate anticoagulation for CVVHDF and such molecule may theoretically interfere with tigecycline plasma protein binding, thus increasing drug-free fraction and extracorporeal removal, the amount of tigecycline clearance was not clinically relevant (14.8% or even smaller due to filter performance decrease over time vs 13% of the total elimination via renal excretion [1]) compared to patients with hypoalbuminemia [2]. Such finding is justified by the lipophilic nature of tigecycline (supported by high volume of distribution [2, 3]), which makes the amount of tigecycline removed negligible and disconnected from plasma protein binding and concentration [4].

However, we support the warning launched by Honore et al. [1] about the potential of molecules’ removal and consequent need for medication dose adjustments when new membranes are used in daily clinical practice. Furthermore, the greater clearance of antibiotics and small molecules associated with high-volume hemofiltration (effluent dose 70 ml/kg/h), as compared with standard volume hemofiltration reported in the IVOIRE study [5], is a matter of concern. Accordingly, we would suggest to titrate drug prescription during renal replacement therapy to specific membrane characteristics and effluent dose delivered to match patient’s need in a timely manner (e.g., acid–base imbalance or fluid overload), especially when hydrophilic medications with low plasma protein binding are prescribed and severe hypoproteinemia occurs [4]. Moreover, we advocate the need for further studies on molecules’ removal by renal replacement therapy and other extracorporeal organ supports, to inform clinicians about the impact of new membranes adsorptive properties, at different effluent doses, on drug pharmacokinetics and patients’ related outcome.

## Data Availability

Not applicable.

## Abbreviations

AN69: Acrylonitrile
CVVHDF: Continuous veno-venous hemodiafiltration
